# Equine bronchial fibroblasts enhance proliferation and differentiation of primary equine bronchial epithelial cells co-cultured under air-liquid interface

**DOI:** 10.1371/journal.pone.0225025

**Published:** 2019-11-13

**Authors:** Vanessa Abs, Jana Bonicelli, Johannes Kacza, Claudia Zizzadoro, Getu Abraham

**Affiliations:** 1 Institute of Pharmacology, Pharmacy and Toxicology, Faculty of Veterinary Medicine, University of Leipzig, An den Tierkliniken, Leipzig, Germany; 2 Saxonian Incubator for Clinical Translation, University of Leipzig, Philipp-Rosenthal-Straße, Leipzig, Germany; 3 Division of Veterinary Pharmacology and Toxicology, Department of Veterinary Medicine, University of Bari, SP 62 per Casamassima, km, Valenzano (BA), Italy; Brigham and Women's Hospital Biomedical Research Institute, UNITED STATES

## Abstract

Interaction between epithelial cells and fibroblasts play a key role in wound repair and remodelling in the asthmatic airway epithelium. We present the establishment of a co-culture model using primary equine bronchial epithelial cells (EBECs) and equine bronchial fibroblasts (EBFs). EBFs at passage between 4 and 8 were seeded on the bottom of 24-well plates and treated with mitomycin C at 80% confluency. Then, freshly isolated (P0) or passaged (P1) EBECs were seeded on the upper surface of membrane inserts that had been placed inside the EBF-containing well plates and grown first under liquid-liquid interface (LLI) then under air-liquid interface (ALI) conditions to induce epithelial differentiation. Morphological, structural and functional markers were monitored in co-cultured P0 and P1 EBEC monolayers by phase-contrast microscopy, scanning and transmission electron microscopy, hematoxylin-eosin, immunocytochemistry as well as by measuring the transepithelial electrical resistance (TEER) and transepithelial transport of selected drugs. After about 15–20 days of co-culture at ALI, P_0_ and P_1_ EBEC monolayers showed pseudo-stratified architecture, presence of ciliated cells, typically honeycomb-like pattern of tight junction protein 1 (TJP1) expression, and intact selective barrier functions. Interestingly, some notable differences were observed in the behaviour of co-cultured EBECs (adhesion to culture support, growth rate, differentiation rate) as compared to our previously described EBEC mono-culture system, suggesting that cross-talk between epithelial cells and fibroblasts actually takes place in our current co-culture setup through paracrine signalling. The EBEC-EBF co-culture model described herein will offer the opportunity to investigate epithelial-mesenchymal cell interactions and underlying disease mechanisms in the equine airways, thereby leading to a better understanding of their relevance to pathophysiology and treatment of equine and human asthma.

## Introduction

Generally, within the pulmonary tissues of all mammalian species including horses, the epithelial and the striking distance to mesenchymal layers of the airway bronchioles provide a physiologically balanced cellular environment and conduct important interactions with the external environment [1]. Airway injuries, which result, for example, from human asthmatic inflammations, structural alterations or foreign particle interaction can markedly hamper airway homeostasis by damaging the structural barrier, which is regulated by epithelial–mesenchymal tight junctions [2–4]. The underlying connective tissue with mesenchymal cells, i.e., fibroblasts, modulates the airway function through extracellular matrix (ECM) deposition and secretion of soluble factors participating in airway inflammation and remodelling [5,6]. With this background in mind, not only epithelial cells can regulate fibroblast proliferation and attraction [7], but also fibroblasts can regulate epithelial cell proliferation, differentiation and wound healing [8–11].

Despite the fact that the equine asthma is the most common disease in adult horses, it has been often hypothesized that allergic disorders may be the consequence of complex interactions between environmental allergens and epithelial tissues and fibroblasts [12]. It is widely accepted that airway inflammation and remodelling are fundamental but the mechanisms that lead to the development of progressive airway obstruction in horses are unclear. Recent investigations suggest that during inflammatory airway disorders extensive injury of the airway epithelium might result in shedding of damaged epithelial cells in the airway lumen but with parallel activation of the remaining surviving epithelial cells and of the underlying fibroblasts in the airways [13–15].

Currently, no experimental work has been done to investigate possible interactions between equine bronchial epithelial cells (EBECs) and equine bronchial fibroblasts (EBFs) that resemble the physical ambient in vivo in airways. Co-culture is a promising alternative to mono-culture and provides a more *in vivo*-like environment for disease and pharmacological studies. The objective of the present study was the first time to evaluate the ability of EBF to direct EBEC proliferation and differentiation as more intricate and controlled in-vivo-like in vitro models. Our aim was to successfully grow and morphologically and functional characterize co-cultured EBECs under air-liquid interface condition. This research work is based on our previously described methods in establishing mono-cultures of EBECs and EBFs [16,17].

## Materials and methods

### Reagents and chemicals

Dulbecco’s Modified Eagle’s Medium (DMEM; with low glucose and L-glutamine), DMEM/Ham´s F12 medium (with 4 mM L-glutamine), Hank’s buffered saline solution (HBSS), phosphate buffered saline (PBS, 0.1 M, pH 7.4), foetal bovine serum (FBS), penicillin, streptomycin and amphotericin B were obtained from Biochrom GmbH (Berlin, Germany). Basal and supplemented Airway Epithelial Cell Growth Medium (AECGM) with its supplement mix was purchased from Promocell GmbH (Heidelberg, Germany). Ultroser-G was obtained from Pall BioSepra (Cergy-Saint-Christophe, France). Atenolol, propranolol, bovine serum albumin (BSA), mitomycin C, retinoic acid, rat tail collagen type I, the rabbit mono-specific antibody (msAb) anti-human tight junction protein 1 (TJP1; also known as zonula occludens (ZO-1; 1:50), goat anti-rabbit IgG (fluorescein isothiocyanate or FITC-labelled, 1:100) and 4',6-diamidino-2-phenylindole-dihydrochloride (DAPI) from Sigma-Aldrich (Deisenhofen, Germany). Mouse monoclonal antibodies (mAbs) anti-human cytokeratins 5/6/18 (clone LP34; 1:10), and anti-bovine vimentin (clone Vim 3B4; 1:10) as well as goat anti-mouse IgG (FITC-labelled; 1:20) were purchased from Dako Deutschland GmbH (Hamburg, Germany).

### Equine bronchial epithelial cells and bronchial fibroblasts

Primary EBECs (n = 7) and EBFs (n = 10) were isolated from the lungs of healthy adult slaughtered horses (median age: 15 years), using our previously described protocols [16–18]. For subsequent assembly of the co-culture system, EBECs isolated from the same donor were used directly as freshly isolated and unpassaged cells (P0) and as sub-cultured cells at passage 1 (P1). The latter were used after growth to 80–100% confluency on collagen-coated tissue culture flasks in the presence of complete AECGM (incl. 10% FBS, 200 U/ml penicillin, 0.2 mg/ml streptomycin and 2.5 μg/ml amphotericin B). EBFs were routinely used at passages between 4 and 8 (P4—P8) which were established from mono-cultures in cell culture flasks using complete DMEM as growth medium (i.e. DMEM supplemented with 10% FBS, 200 U/ml penicillin, 0.2 cm/ml streptomycin and 2.5 μg/ml amphotericin B).

### Non-cell-cell contact EBEC-EBF co-culture

Since fibroblasts quickly proliferate and can be used in co-culture only for quite few days, it was necessary to arrest proliferation and growth of these cells before using them in co-culture with EBECs. Accordingly, first, EBFs were seeded at a density of 10^4^ cells/cm^2^ in 24-well plates (Greiner, Frickenhausen, Germany) and grown in complete DMEM for 3 days to achieve ~ 80% confluency. Then, cells were treated with mitomycin C (10 μg/ml for 8 hours). Treated EBF monolayers were washed with PBS to remove mitomycin C and fresh medium (complete DMEM) was added, and allowed to stabilize for 3 hours. Thereafter, uncoated semi-permeable membrane inserts (PET; 0.4 μM pore size; Greiner Bio-One, Frickenhausen, Germany) were placed into the wells which already contain mitomycin C-treated EBF monolayers, and then unpassaged (P_0_) or passaged (P_1_) EBECs in complete AECGM were plated apical on the upper surface of inserts at a density of 0.9 x 10^6^ viable cells/cm^2^. After so establishing the liquid-liquid interface (LLI) condition, the medium in the basolateral chamber (1 ml complete DMEM) and that in the apical compartment (0.3 ml complete AECGM) were changed initially every 24 hours post-EBEC seeding and then, every 48 hours until EBEC monolayers reached full confluency. Then, an air-liquid interface (ALI) condition was created (day 0) by removing the medium from the apical compartment and by replacing the basolateral medium with ALI-medium. DMEM/Ham’s F12 medium was used as ALI medium (supplemented with 2% ultroser-G, 15 ng/ml retinoic acid, 200 U/ml penicillin, 0.2 ng/ml streptomycin and 2.5 μg/ml amphotericin B), which was changed every 2–3 days over a 60-day period of observation. The degree of differentiation of co-cultured P0 and P1 EBEC monolayers was evaluated by assessing an array of specific morphological, structural, ultrastructural and functional markers at selected time points or routinely as detailed below.

### Light microscopy

EBEC-EBF co-cultures were monitored routinely to assess morphological features (confluency and integrity of cell layers, cell shape, EBEC ciliary beating, EBEC mucus secretion) using bright field phase-contrast inverted CKX41 microscope (Olympus Optical Co. Ltd., Tokyo, Japan) equipped with a color DD60 U-PM TVC Canon digital camera.

### Histological analysis

At selected time points, membrane inserts with confluent monolayers of co-cultured P0 and P1 EBECs at LLI or ALI were washed with PBS, fixed in 4% paraformaldehyde, embedded in paraffin, sectioned and processed by standard hematoxylin/eosin (H&E) staining to evaluate the structure and composition of the epithelium.

### Ultrastructural analysis

Membrane inserts with co-cultured EBECs at ALI were removed from membrane holding devices and washed twice with PBS. Each sample was then fixed in 4% (v/v) paraformaldehyde in 0.1 M PBS (Histofix; Carl Roth GmbH, Karlsruhe, Germany) overnight at 4°C and postfixed in 1% phosphate-buffered osmium tetroxide for 1 hour at room temperature. After three times washing with PBS, the membranes were dehydrated in a graded series of ethanol (30–100%) and dried with a critical point dryer (CPD 030, BAL-TEC, Witten, Germany) and stored on silica gel orange (Carl Roth GmbH, Karlsruhe, Germany). Prior to scanning electron microscopy (SEM), specimen were sputter-coated with Gold-Palladium (90/10) at a specimen-target distance of 50 mm with approximately 40 mA for 60 seconds (MED 020, BAL-TEC, Liechtenstein) and viewed by scanning electron microscope (SEM) (LEO 1430 vp; Zeiss NTS (Oberkochen, Germany).

In parallel, membrane inserts were also processed for transmission electron microscopy (TEM) to assess the integrity of the inner structure of co-cultured EBECs. After washing with 0.1 M PBS (pH 7.4), cells were fixed with a mixture of 2.5% glutaraldehyde and 4% of paraformaldehyde in PBS overnight at 4°C. Membranes were cut into pieces (4 x 4 mm) and postfixed in phosphate-buffered 1% osmium tetroxide at 4°C for 2 hours and then dehydrated in graded series of ethanol and embedded in plastic resin, Glycidether100^TM^ (Carl Roth GmbH, Karlsruhe, Germany). Ultrathin sections were cut with Leica Ultracut UCT (Leica Microsystems, Wetzlar, Germany) and mounted on 300 mesh thin bar nickel grids. Specimens were viewed on EFTEM Libra 120 (Zeiss NTS, Oberkochen, Germany). Digital images were captured using a Sharpeye 2k CCD camera (TRS, Moorenwies, Germany).

### Fluorescence microscopy

At different time points, membrane insert samples with confluent monolayers of co-cultured P0 and P1 EBECs at LLI and ALI were immunostained for specific marker proteins (epithelial cell marker cytokeratin, mesenchymal cell marker vimentin, TJP1) to confirm their epithelial nature and purity, as well as to check the expression of intercellular tight junctions. At the same time, glass cover slips with mytomicin C-treated monolayers of co-cultured EBFs in ALI-medium were immunostained to confirm their mesenchymal nature and purity. Briefly, membrane inserts and cover slips were washed with PBS and fixed in ice-cold acetone (for cytokeratin and vimentin detection) or methanol (for epithelial tight junction protein 1 (TJP-1 or ZO-1) detection) for 5–10 min at -20°C. Samples were then washed in PBS, neutralized in 50 mM NH_4_Cl in PBS for 10 min at room temperature (RT). Nonspecific antibody binding was reduced by incubation in 3% (w/v) bovine serum albumin (BSA) solution overnight at 4°C. Samples were then incubated with the primary antibodies for 1 hour at RT. Protein expression was visualized with appropriate FITC-labelled secondary antibodies and DNA staining using DAPI visualized nuclei. Immunostained cell preparations were mounted with antifade Fluoromount-G mounting medium^r^ and fluorescent labelling was visualized using a BX50 fluorescence microscope (Olympus) and image acquisition software.

### Cell barrier integrity analysis

During co-culturing of EBECs onto transwell membrane inserts in the growth medium at LLI or ALI conditions, the integrity of cell layers and the development of tight junctions were examined by the transepithelial electrical resistance (TEER) measurements using an EVOM voltohmmeter (Millicell^®^-ERS, Millipore Corp., Billerica, MA, USA) as previously described [16]. The apical and basolateral chambers were filled with fresh medium and inserts with the cells were pre-equilibrated before TEER measurement for 30 min 37°C in a humidified atmosphere with 5% CO_2_. In EBEC-EBF co-cultures at ALI, the medium was removed from the apical compartment immediately after measurement. The TEER values of the cell layers were corrected by subtracting the background TEER values measured in the inserts without cells with media in both chambers and the area of the porous insert membranes (0.336 cm^2^) was considered in the TEER units (Ω*cm^2^). Data are expressed as mean ± standard error of the mean (S.E.M.).

### Drug transport studies

Co-cultured monolayers of P0 EBEC at ALI were examined for the development of the selective permeability properties related to tight junction formation and cell layer integrity by studying the apical-to-basolateral transeipthelial transport of two β-receptor antagonists. The hydrophilic drug atenolol was used to confirm cell layers’ integrity on the porous membranes as a paracellular marker, and the lipophilic drug propranolol as a passive transcellular marker. In brief, confluent primary EBEC (P_0_) co-cultures were cultivated over several days (7, 9 and 11 days) on transwell membrane inserts under LLI or ALI condition. TEER across cell layers was measured before initiating the transport study by replacing the medium in the apical compartment with test compounds dissolved in Hank´s buffered saline solution (HBSS), and was above the threshold value of 300 Ω*cm^2^. Membrane inserts with EBEC monolayers were removed from co-cultures and transferred into new empty transwell plates. Fresh pre-warmed HBSS was then added to both apical and basolateral compartments and cell monolayers were allowed to equilibrate in the transport medium for 15 min at 37°C in a humidified atmosphere with 5% CO_2_. Thereafter, HBSS was removed and flux was initiated by adding atenolol and propranolol (100 μM each) into the apical side (donor) and drug-free HBSS to the basolateral compartment (acceptor). At incubation time points of 10, 30 and 50 min, transport studies were performed on a shaking platform at 37°C in a humidified atmosphere with 5% CO_2_. Triplicate samples (each 10 μl) were collected from the basolateral chamber (acceptor side) at the indicated time points after drug application to the donor compartment and the original volume was replaced by adding equal amount of HBSS (10 μl). At the end of each experiment, the integrity of tight junctions of co-cultured EBEC monolayers was confirmed by measuring TEER, and then calculating the percentage changes relative to values measured prior to the experiment. Both compounds were then analyzed by liquid chromatography tandem mass spectrometer (LC-MS/MS). The apparent permeability coefficient of each marker compound (P_app;_ cm/s) was then calculated according to the equation: P_app_ = (dQ/dt)*(1/AC_0_), where dQ/dt is the transport rate of the drug across the cell monolayer, A the surface area available for transport and C_0_ the initial drug concentration in the donor compartment.

All transport experiments were performed in triplicates (i.e. using three monolayers for each EBEC isolate) and permeability data are expressed as mean ± S.E.M. (of 5 different EBEC isolations). The effect of co-culturing EBECs with EBFs vs. control (blank) on drug transport at different culturing period was compared using two-way ANOVA (GraphPad Prism software, version 6.0, CA, USA). All P values of less than 0.05 were considered significant.

## Results

### Proliferation and morphological studies of P0 and P1 EBEC in co-culture with EBFs

**Fig 1:**
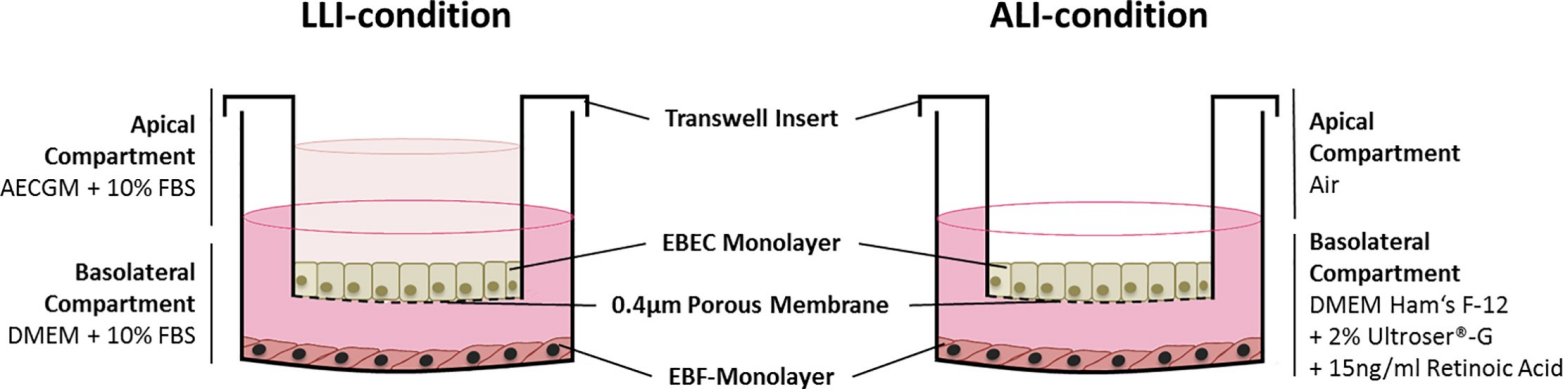
Schematic representation of the EBEC-EBF transwell co-culture model under liquid-liquid (LLI) and air-liquid interface (ALI) condition. Equine bronchial fibroblasts (EBFs) at passages between 4 and 8 were seeded on 24-well plates and treated with mitomyin C when they reached 80% confluency. Freshly isolated (P0) or passaged (P1) EBECs were seeded on membrane inserts that were placed inside the EBF-containing well plates. EBECs were first allowed to grow under liquid-liquid interface (LLI) conditions (with complete DMEM in the basal compartment and complete AECGM in the apical compartment) until they formed confluent monolayers, then air-liquid interface (ALI) conditions (with ALI medium in the basal compartment and air in the apical compartment) were established to induce epithelial differentiation.

**Fig 2:**
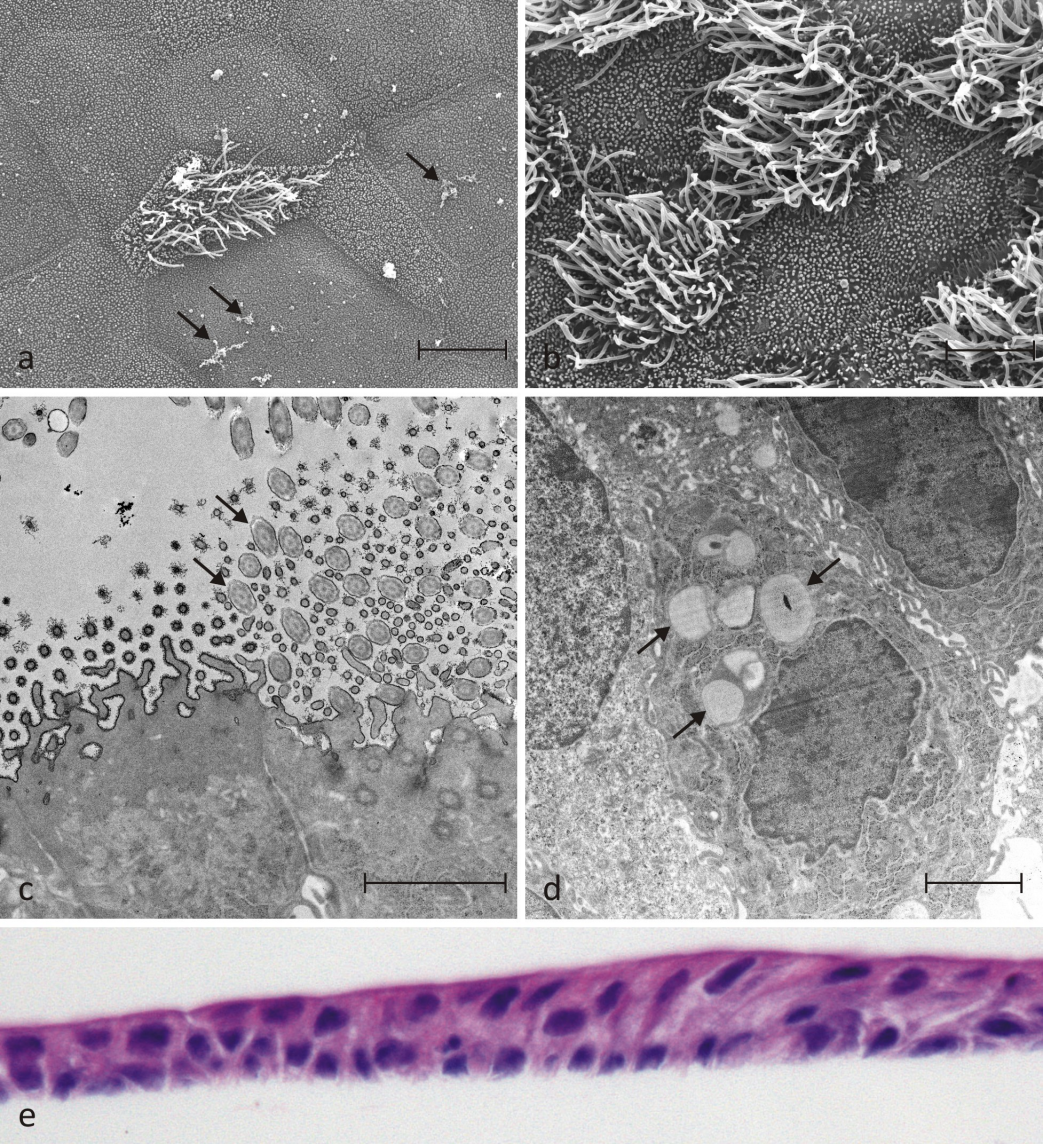
Phenotypic features of morphological, structural and ultrastructural differentiation detected in P0 and P1 EBEC monolayers during co-culture with mitomycin C-treated EBFs under ALI-conditions. Representative SEM micrographs revealing the presence of cilia after 8 days (a) and 20 days (b) of co-culture at ALI, as well as of microvilli (a, b) and mucus-like amorphous material (a) (arrows). TEM micrographs showing cross sections of a number of cilia (c) with typical internal structures of their basal body or centriole (the so called 9+0 arrangement of microtubules, consisting of a circle of 9 sets of triplets embedded in the apical part of the epithelial cells; black arrow heads) and motile part (the so-called 9+2 arrangement of microtubules, consisting of a circle of 9 doublets and one central doublet; black arrows), cross sections of a number of microvilli and a mucus-containing cell (d, black arrows). Representative images of H&E- (e) stained sections of “matured” epithelial layers (15–20 days of ALI) showing pseudo-stratified columnar architecture (e; magnification: x40). The scale bar for (a) was 10 μM, for (b) 5 μM as well as for both c and d 2 μM, respectively.

**Fig 3:**
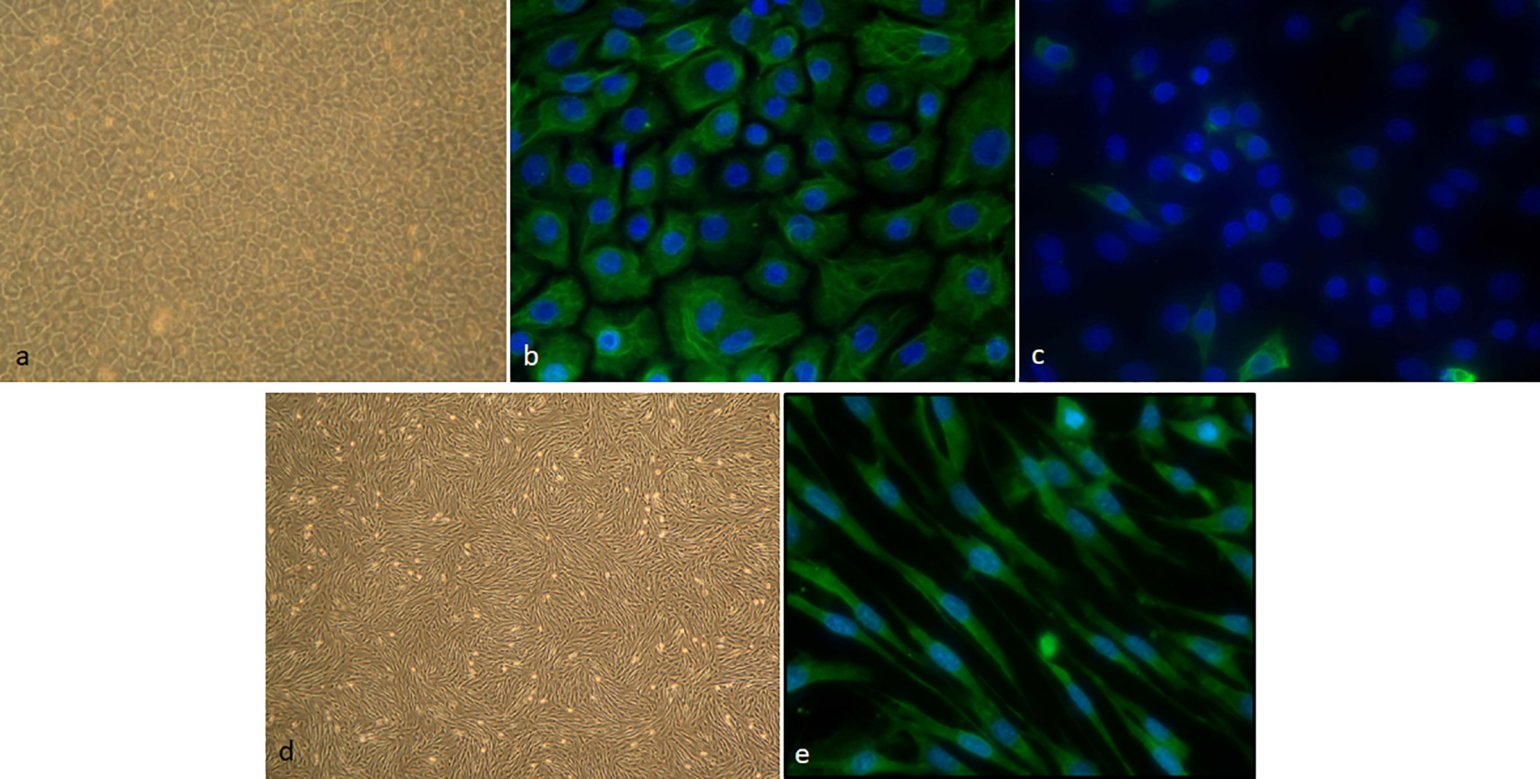
Purity of EBEC and EBF monolayers in co-culture under ALI conditions as depicted in Fig 1. Representative phase-contrast microscopy images [magnification: x4] showing the morphological appearance of “mature” confluent P1 EBEC monolayers after 15–20 days of co-culture at ALI (a; P_1_) and of 80% confluent monolayers of mitomycin C-treated EBFs maintained in ALI-medium for the same culture period (d; P6)). Representative images of immunofluorescence staining of confluent P1 EBEC monolayers after 15–20 days of co-culture at ALI for the epithelial cell marker cytokeratin (b P0; and c nuclei blue stained with DAPI) and the mesenchymal cell marker vimentin (rest of green fluorescence in EBEC P0 c; e) [magnification: x40; green fluorescence (FITC) is positive signal; nuclei are blue stained with DAPI].

A schematic representation of how EBECs and mitomycin C-treated EBFs were assembled in the co-culture system is given in Fig 1. When seeded on the upper surface of the uncoated transwell inserts, more than 96% of the P0 and P1 EBECs attached within the first 24 hours (P0: 96.8 ± 4.1%, S.E.M., n = 4; 97.1 ± 2.8%, S.E.M., n = 4). While P0 EBECs maintained under LLI conditions, they formed dense confluent monolayers within 3–5 days, whereas P1 EBEC monolayers needed about one week (5–10 days) to be confluent. The appearance of significant morphological, structural and ultrastructure features of differentiation was observed in the co-cultured epithelial layers after establishment of ALI condition. Electron microscopic examination of P0 and P1 EBEC monolayers revealed the presence of typically structured cilia and microvilli on the cell apical surface starting from day 7 of co-culture at ALI (Fig 2A and 2B), with the proportion of ciliated cells increasing with increasing days of ALI (Fig 2B). TEM and SEM also detected mucus producing goblet-like cells and droplets of mucus-like materials within the first week of ALI (Fig 2C and 2D). In “mature” P0 and P1 EBEC monolayers, after about 15–20 days of co-culture at ALI, histological and histochemical examination revealed the formation of pseudo-stratified columnar epithelium, consisting of a mixed population of basal and differentiated cells, the latter including both pseudo-stratified and ciliated (Fig 2E). Besides the polygonal cell shape with typical cobblestone appearance (Fig 3A) (P1), immunofluorescence staining for specific epithelial and mesenchymal marker proteins confirmed the dominant epithelial nature of all “mature” monolayers of co-cultured P0 and P1 EBECs at ALI which usually consisted of more than 95% cytokeratin positive cells (Fig 3B; P0) and less than 5% vimentin-positive cells (Fig 3C; EBEC P0). Parallel microscopic observation of monolayers of mitomycin C-treated EBFs co-cultured in ALI-medium with P0 and P1 EBECs revealed uniform cell populations with typically elongated spindle shape (Fig 3D; EBF P6), that consistently proved > 95% vimentin-positivity at immunofluorescence (complete absence of cytokeratin-positive epithelial cells) (Fig 3E, P6).

### Effect of epithelial exposure to EBF on epithelial integrity

**Fig 4:**
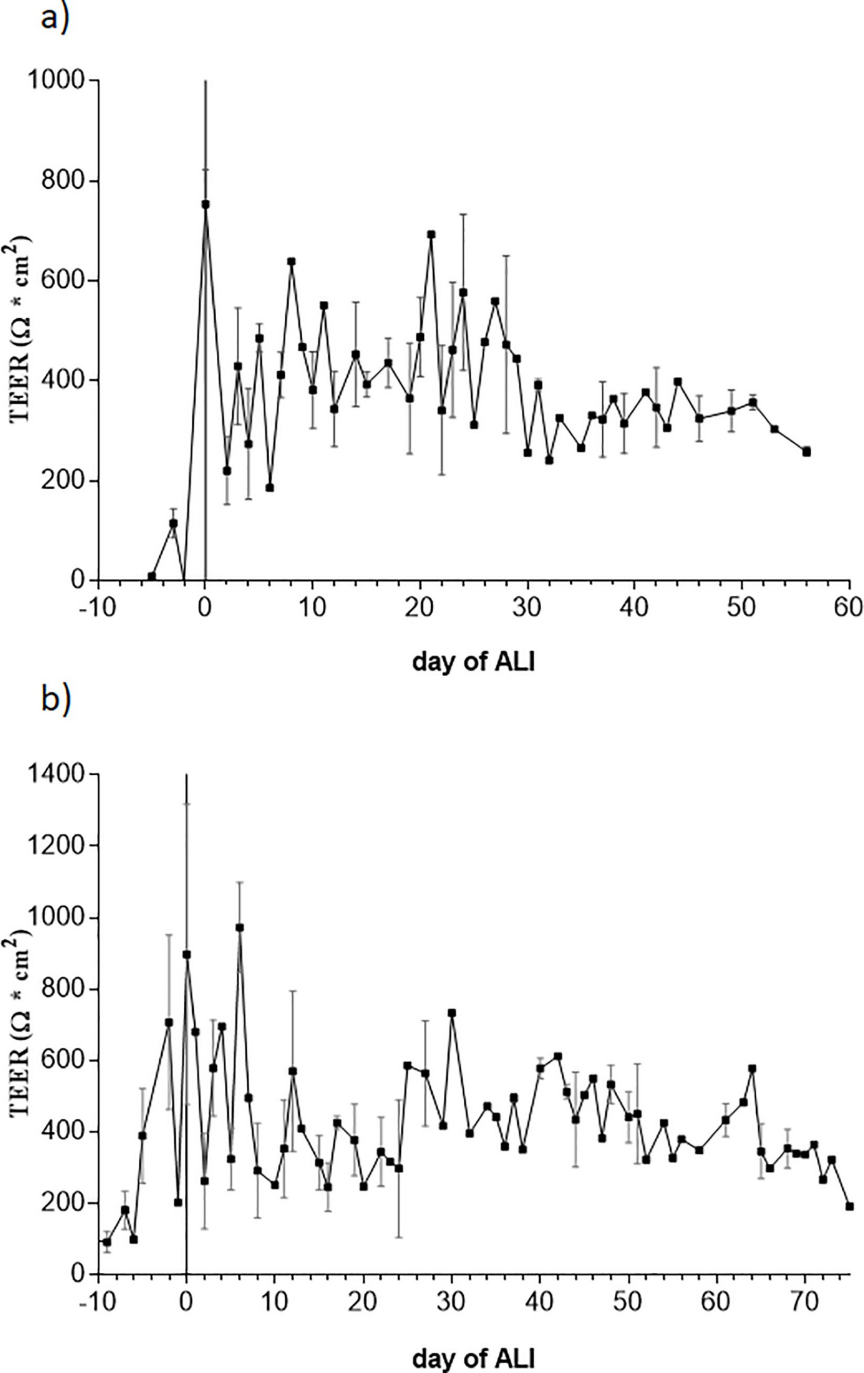
Bioelectrical characteristics of EBECs grown on insert membranes in co-culture with mitomycin C-treated EBFs. The graphs show the time-course of TEER development in P0 (a) and P1 (b) co-cultured EBEC monolayers. The abscissa indicates co-culture days before (negative) and after (positive) switch to the ALI condition (day 0). Each point represents mean ± SEM (n = 5, except for the TEER graph of P1 EBECs from ALI day 20 to ALI day 60, where n = 3).

Differentiation capacity was further confirmed by measuring transepithelial electrical resistance (TEER) and tight junction formation. A measurable TEER was constantly detected in both P0 and P1 EBEC monolayers, confirming the ability of these cells to form functional tight junctions under the co-culture conditions adopted (Fig 4). Under LLI conditions, TEER increased in parallel with cell proliferation, reaching mean values of 753 Ω*cm^2^ (± 70; n = 5) and 663 Ω*cm^2^ (± 265; n = 5) in confluent monolayers of co-cultured P0 and P1 EBECs, respectively. When switching to ALI (day 0), TEER of co-cultured P0 EBECs dropped abruptly reaching about 40% of its initial value, and this was usually accompanied by the appearance of holes in the monolayer. The integrity of the latter was completely restored within about 5 days of co-culture at ALI, with parallel gradual increase in TEER to a stable value of 314.4 Ω*cm^2^ (± 250.3, n = 5) which was achieved by days 10–15 of ALI and remained stable at least until ALI day 30 (Fig 4A). Confluent monolayers of co-cultured P1 EBECs did not lose their morphological integrity when switched to the ALI condition. The TEER value was only slightly affected by this event, with the achievement of a stable value of about 400 Ω*cm^2^ (502.2 Ω * cm^2^ ± 250.6, n = 5) by ALI day 7. Then, after ALI day 20, TEER values started to decline irreversibly in the monolayers from two subjects, but were maintained on a slightly higher level until ALI day 50 in the monolayers from the other three subjects (495.3 Ω * cm^2^ ± 350.2, n = 3) (Fig 4B).

**Fig 5:**
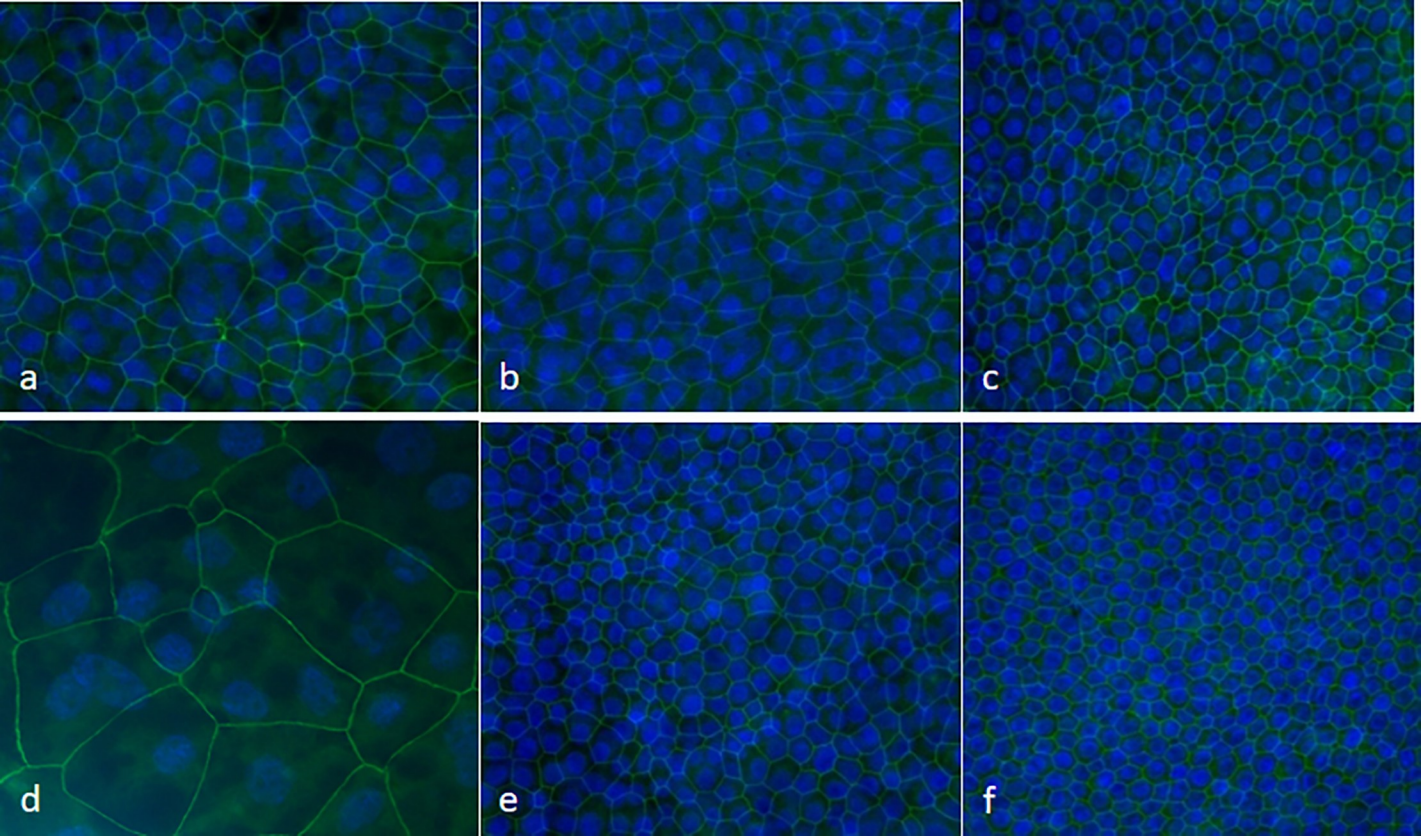
Time-course of tight junction expression pattern in EBEC monolayers grown in the presence of monolayers of mitomycin C-treated EBFs. Representative images of confluent P_0_ (a-c) and P_1_ (d-f) EBEC monolayers immunolabelled for TJP1 (green fluorescence) immediately before switching from the LLI to the ALI condition (ALI day 0) (a, d), shortly after the achievement of a stable TEER value under ALI conditions (P0: ALI days 10–15; P1: ALI days 7–10 days) (b, e) and in more “mature” co-cultures (ALI days 15–20) (c, f). Magnification: x40; nuclei are blue stained with DAPI.

The expression of the tight junction proteins responsible for the bioelectrical properties of the co-cultured epithelial monolayers was confirmed by immunofluorescence. Confluent monolayers of both P0 and P1 co-cultured EBECs showed positive continuous staining for TJP1 around the periphery of each cell. Under ALI conditions, the number of TJP-1 positive cells per field increased gradually with the age of the co-culture, with "mature" co-cultured EBEC monolayers (i.e. at 15–20 days of ALI) showing very small and tight TJP-1 fluorescent rings that could hardly be captured on the same plane of focus of the nuclear stains (Fig 5).

### Drug permeability across P0 EBECs in co-culture with EBFs at ALI

During the transport experiments TEER of EBEC monolayers was not affected, which remained after completion of each experiment at about 100% of the TEER measured at the beginning, confirming tolerability of the experimental conditions adopted for the study.

**Fig 6:**
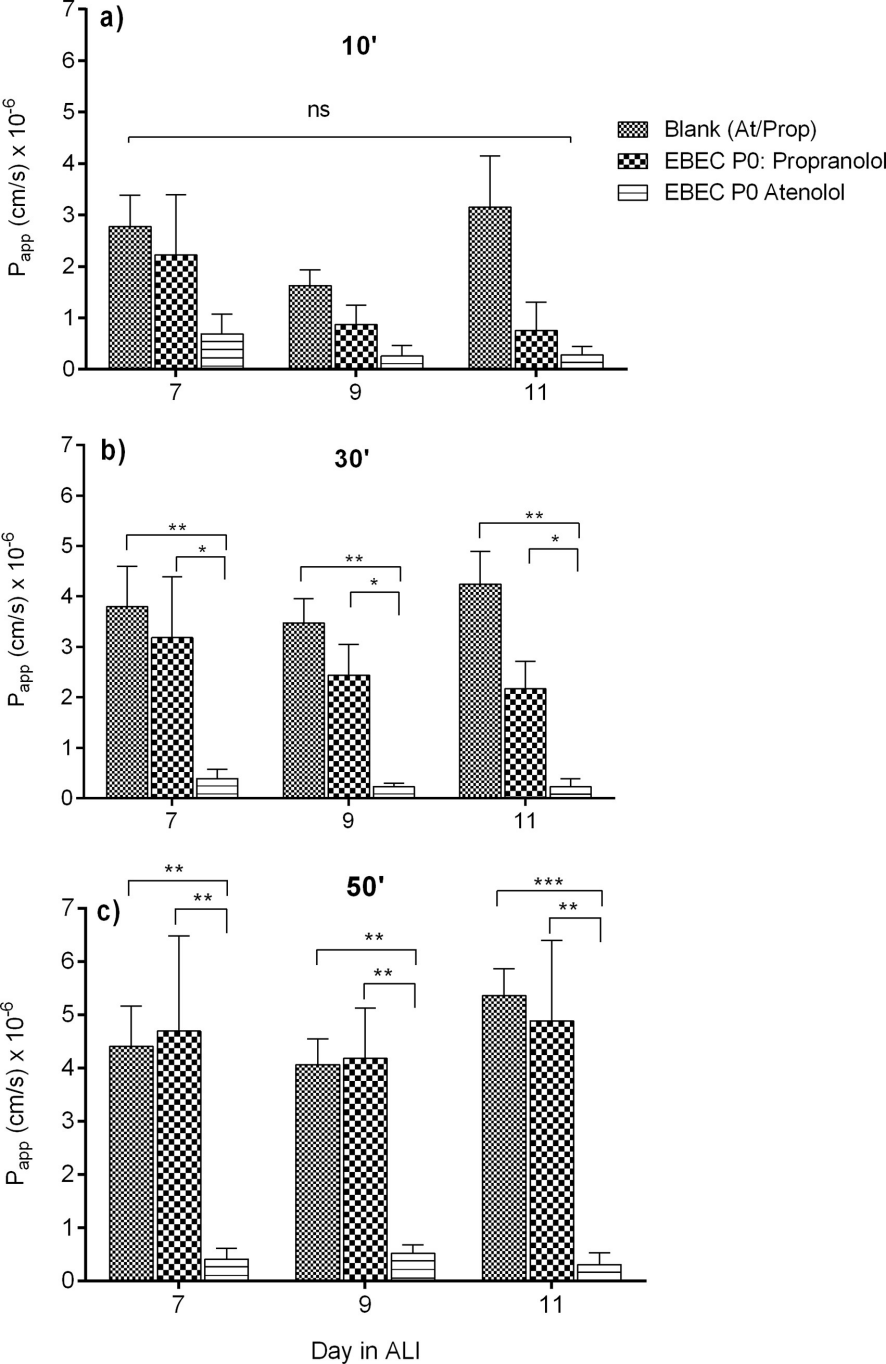
The apparent permeability coefficients (P_app_) for the apical-to-basolateral transport of atenolol and propranolol across P0 EBEC monolayers co-cultured under ALI conditions with mitomycin C-treated EBFs. The graph compares the mean (± SEM) permeability values (P_app_) calculated for the paracellular transport marker atenolol and the transcellular transport marker propranolol with time in co-culture P0 EBEC monolayers at ALI for 7, 9 and 11 days (n = 5) and across blank (cell-free) inserts (n = 5) after 10 (a), 30 (b) and 50 (c) min of incubation. Data are depicted as means ± S.E.M from n = 5–8 independent experiments. Asterisks indicate statistical significance from blank values and between drugs; *(p<0.05), **(p<0.01), ***(p<0.001) and ns: not significant.

As depicted in Fig 6, for both marker compounds, the cumulative amount of drug measured in the acceptor compartment increased in a time-dependent manner and with linear rate over the time course of 10 over 50 min in each co-culture day of 7, 9 and 11, consistent with passive diffusion. However, the P_app_ value calculated for the paracellular transport marker atenolol across all 7-, 9- and 11-day-old co-cultured P0 EBEC monolayers at ALI and at all time-points was decreasing compared to the transcellular marker propranolol under the same conditions. After 30–50 min of incubation and at all days of co-culture, the atenolol transport was approximately 10–16 times significantly lower than propranolol. That means, the paracellular permeability of atenolol decreased with time in culture as the cell layers developed a concomitant increase in TEER (Fig 4).

## Discussion

Despite the fact that in equine and human asthma epithelial-fibroblast cell interactions are crucial in airway wall thickening and disease morbidity, only few studies have been undertaken investigating the interaction of these airway cells *in vitro*. To the Authors' knowledge, the present paper is the first to describe a co-culture model where a monolayer of primary EBECs is grown on a porous transwell insert at an ALI in the presence of an underlying monolayer of primary EBFs grown on culture well plates. The interactions occurring between the epithelium and adjacent mesenchyme are thought to play essential role in many key homeostatic processes of the airways (such as normal repair) that become dysfunctional in disease states such as asthma [19,20], and co-cultures of airway epithelial cells and fibroblasts are particularly valuable tools for research in this area [8].

There are multiple options on how to design a co-culture model of these two cell types. Some setups involve direct cell-cell contact and their most popular version is known as "feeder layer" system, because the airway epithelial cells are seeded and grown directly on the top surface of previously established fibroblast monolayers [8,10]. Other setups prevent physical contact between the two cell populations, but permit the exchange of soluble factors and are, therefore, indicated as "paracrine" co-culture systems. This condition is achieved by interposing a permeable membrane. In most cases, this serves as the support on which the epithelial cells are grown while fibroblasts are instead grown either on the underside of the same membrane (i.e. in close proximity) or at the bottom of the culture well plates on which the epithelial cell bearing membrane is usually held by an insert [8,10,21,22]. Each option has advantages and limitations, and allows obtaining only definite information: the choice depends often on the experimental question being addressed by the co-culture study [23,24].

Our EBEC-EBEF co-culture model has been designed as a versatile and manipulable two-chamber system in which the two cell types are grown on distinct and segregable culture supports, without any direct cell-cell contact and not in close proximity, but sharing a common aqueous environment through which cells can communicate via the release of soluble mediators. The model is, therefore, particularly suitable for studying the reciprocal modulation of either epithelial cell or fibroblasts functions through paracrine signalling. The model should enable researcher on-demand separation of the individual cell populations, thereby allowing dissection of the spatial and temporal release of the mediators. Moreover, it offers the potential to identify the key cell type-specific mediators in the co-culture media and define the molecular mechanisms (receptors and signaling pathways) that affect cells in co-culture [23].

In our model, EBFs were treated with mitomycin C to inhibit their proliferation. In a separate series of experiments, we attempted to establish the co-culture by using normal proliferating EBFs. However, these cells could not be maintained in co-culture with the EBECs for a sufficiently long period of time (necessary for EBECs to achieve full differentiation), because once having formed a confluent monolayer, they remained viable for only two weeks, after which they became senescent and detached. The use of mitomycin C as a strategy to overcome this problem was described by Skibinski et al. [8] who treated human airway fibroblasts and found that these cells, although totally and irreversibly unable to proliferate, were able to survive in culture medium as attached cells for up to 6 weeks. The response of EBFs to mitomycin C in our study was similar to that reported for human cells, as the life span of post-mitotic EBFs extended over the entire period of EBEC co-cultures. Based on this finding, it might be assumed reasonable to infer that the response of EBFs to mitomycin C was similar to that reported for human airway fibroblasts. The fact that treatment with this substance did not affect their secretory function, it can be expected that post-mitotic EBFs were able to constitutively express cytokines and to react to external stimuli (including soluble mediators of epithelial origin) by modulating their mRNA and protein expression, just as it has been shown to occur in the human-derived cells [8].

Furthermore, our study strongly suggest that the post-mitotic EBFs in our co-culture system do exhibit a secretory phenotype and that the soluble substances they release into the basolateral medium are somehow supportive to the growth and differentiation of EBECs. These elements become evident when the protocol we previously developed in our lab for generating differentiated EBEC monocultures on transwell inserts at ALI [16] is considered for comparison. This can be summarized as follows: first, in contrast to the previous work, when assembling the co-culture system, there was no need to coat the transwell inserts with an extracellular matrix component (e.g. collagen) for EBECs to attach efficiently (> 96%). On the other hand, collagen coating of inserts was a prerequisite for successful establishment of EBEC mono-cultures. Second, both P0 and P1 EBEC co-cultures could be routinely seeded on inserts at the same density (0.9 x 10^6^ viable cells/cm^2^) which is low when compared to the cell densities that proved necessary to generate P0 EBEC mono-cultures (often > 1 x 10^6^ viable cells/cm^2^) and P1 EBEC mono-cultures (even > 2.2 x 10^6^ viable cells/cm^2^). Third, P1 EBEC co-cultures at LLI were able to form confluent and tight monolayers in about one week (5–10 days), while mono-cultures of P1 EBECs usually were far from confluence and consistently showed rather low TEER values (50–100 Ω*cm^2^) even after 12 days of culture under LLI conditions [16]. Fourth, monolayers of P0 EBEC co-culture were affected by switching to the ALI condition (e.g. hole appearance) and took just 5 days on average to restore their morphological and functional integrity. A complete recovery of P0 EBEC mono-cultures usually required no less than 18–21 days of ALI. Fifth, monolayers of P1 EBEC co-cultures started to show morphological signs of differentiation (particularly the presence of cilia) after just 7 days of ALI, while the appearance of ciliated cells in mono-cultures of P1 EBECs was usually observed at around day 30 of ALI.

On the basis of human-derived data of airway cells [8, 25], it can be speculated that the soluble substances released by mitomycin C-treated EBFs in our co-culture system include members of the FGF family of cytokines, which are known to variably exert positive influence on the adhesion, growth and/or differentiation of different types of epithelial cells, including those of the airways. However, the identification of the putative EBF-derived paracrine mediators was beyond the scope of our study and will certainly represent the subject for future investigations. It also remains unknown whether the release of these substances by EBFs occurs spontaneously or in response to some specific signals released by the EBECs immediately after seeding in the co-culture system. Experiments using conditioned media obtained from mono-cultures of the individual cell types will likely help answer this question. Furthermore, by comparing co-cultures assembled with epithelial cells and fibroblasts from healthy and asthma-affected horses, it will be possible to identify the regulatory mechanisms of airway cell cross-talk that are dysfunctional in disease.

In light of all these potential future developments, it is worth noting that in our co-culture model fibroblasts were exactly from the same anatomical site as the epithelial cells (i.e. bronchial wall). Indeed, there is evidence that epithelial-fibroblast paracrine interactions take place in a qualitative and quantitative different manner depending on the specific organ [26]. Other authors reported that fibroblasts from different body sites (such as lymph nodes or skin) can be less effective than airway fibroblasts in supporting airway epithelial cell growth and differentiation in co-culture, presumably due to different patterns of cytokine release [8,21].

As described above, the overall positive influence of EBFs on EBECs in our co-culture system could mainly be observed with respect to the rate of epithelial cell growth and differentiation. As for the final degree of morphological and functional differentiation that co-cultured EBEC monolayers were able to achieve under ALI-conditions, no relevant differences could be noted when compared with the pattern of differentiation characterized previously for P0 and P1 EBEC mono-cultures under ALI [16]. Indeed, during ALI, both P0 and P1 EBEC co-cultures acquired a polarized phenotype and formed a ciliated pseudo-stratified epithelium, endowed with barrier properties that are typically determined by the presence of functional intercellular tight junctions that include the ability to discriminate between the permeability of lipophilic and hydrophylic compounds, with effective restriction of paracellular transport across the cell monolayer. These data, in the first place, confirm the critical importance of using air-liquid interface conditions for promoting the proper differentiation of primary airway epithelial cells in vitro; second, they indicate, that the established co-culture conditions could improve the proliferation of EBECs, without altering their differentiation ability.

In summary, this study provides the scientific community a co-culture model of primary equine bronchial epithelial cells and fibroblasts, which allows a quite realistic representation of the physiologic microenvironment of the equine bronchial wall. This helps assessing the exposure of a morphologically and functionally differentiated epithelium to inhaled air and airborne particles, chemicals and pathogens, to an aqueous medium containing a milieu of soluble mediators that allow epithelial cells and fibroblasts of the underlying mesenchyme to exchange information and coordinate their functions, in order to maintain tissue homeostasis and restore balance after perturbation. By enabling mechanistic investigations of the paracrine signalling underlying this bidirectional communication, the use of this model offers the possibility to gain new insights into the pathophysiology and treatment of equine asthma and its human disease counterpart.

Model limitations: The establishment of functional polarity, cellular phenotypic stability and the demonstration of enhanced induction of proliferation and integrity of tight junction barrier clearly represent important advances in the development of equine airway epithelial co-culture models, indeed, with limitations. For example, the role of specific paracrine signalling in the system has not been evaluated from both sides of the cells. It is also acknowledged in the current study that we have not addressed the critical role of soluble factors of both cell types in the co-cultures, and this represents an important and highly relevant element missing at this time. Ongoing efforts in our lab focus on overcoming these technical challenges to enhance the efficiency of the model.

## References

[1] Holgate ST, Lackie PM, Davies DE, Roche WR, Walls AF. The bronchial epithelium as a key regulator of airway inflammation and remodelling in asthma. Clin Exp Allergy 1999; 29 Suppl. 2: 90–95. 10421830 10.1046/j.1365-2222.1999.00016.x

[2] Xiao C, Puddicombe SM, Field S, Haywood J, Broughton-Head V, Puxeddu I, et al Defective epithelial barrier function in asthma. J Allergy Clin Immunol. 2011; 128: 549–556. 10.1016/j.jaci.2011.05.038 21752437

[3] Humlicek AL, Manzel LJ, Chin CL, Shi L, Excoffon KJ, Winter MC, et al Paracellular permeability restricts airway epithelial responses to selectively allow activation by mediators at the basolateral surface. J Immunol. 2007; 178: 6395–6403. 10.4049/jimmunol.178.10.6395 17475869

[4] Coyne CB, Vanhook MK, Gambling TM, Carson JL, Boucher RC, Johnson LG. Regulation of airway tight junctions by proinflammatory cytokines. Mol Biol Cell. 2002; 13: 3218–3234. 10.1091/mbc.E02-03-0134 12221127 PMC124154

[5] Haag S, Matthiesen S, Juergens UR, Racké K. Muscarinic receptors mediate stimulation of collagen synthesis in human lung fibroblasts. Eur Respir J. 2008; 32: 555–562. 10.1183/09031936.00129307 18480105

[6] Wenzel SE, Trudeau JB, Barnes S, Zhou X, Cundall M, Westcott JY, et al TGF-beta and IL-13 synergistically increase eotaxin-1 production in human airway fibroblasts. J Immunol. 2002; 169: 4613–4619. 10.4049/jimmunol.169.8.4613 12370400

[7] Malavia NK, Mih JD, Raub CB, Dinh BT, George SC. IL-13 induces a bronchial epithelial phenotype that is profibrotic. Respir Res. 2008; 9: 27 10.1186/1465-9921-9-27 18348727 PMC2292179

[8] Skibinski G, Elborn JS, Ennis M. Bronchial epithelial cell growth regulation in fibroblast cocultures: the role of hepatocyte growth factor. Am J Physiol Lung Cell Mol Physiol. 2007; 293: L69–L76. 10.1152/ajplung.00299.2006 17384084

[9] Myerburg MM, Latoche JD, McKenna EE, Stabile LP, Siegfried JS, Feghali-Bostwick, CA, et al Hepatocyte growth factor and other fibroblast secretions modulate the phenotype of human bronchial epithelial cells. Am J Physiol Lung Cell Mol Physiol. 2007; 292: L1352–L1360. 10.1152/ajplung.00328.2006 17307814

[10] Wiszniewski L, Jornot L, Dudez T, Pagano A, Rochat T, Lacroix JS, et al Long-term cultures of polarized airway epithelial cells from patients with cystic fibrosis. Am J Respir Cell Mol Biol. 2006; 34: 39–48. 10.1165/rcmb.2005-0161OC 16179582

[11] Werner S, Smola H. Paracrine regulation of keratinocyte proliferation and differentiation. Trends Cell Biol. 2001; 11: 143–146. 10.1016/s0962-8924(01)01955-9 11306276

[12] Barton AK, Gehlen H. Pulmonary Remodeling in Equine Asthma: What Do We Know about Mediators of Inflammation in the Horse? Mediators Inflamm. 2016; 2016: 5693205 10.1155/2016/5693205 28053371 PMC5174180

[13] Bullone M, Lavoie JP. Asthma "of horses and men"—how can equine heaves help us better understand human asthma immunopathology and its functional consequences? Mol Immunol. 2015; 66: 97–105. 10.1016/j.molimm.2014.12.005 25547716

[14] Setlakwe EL, Lemos KR, Lavoie-Lamoureux A, Duguay JD, Lavoie JP. Airway collagen and elastic fiber content correlates with lung function in equine heaves. Am J Physiol Lung Cell Mol Physiol. 2014; 307: L252–L260. 10.1152/ajplung.00019.2014 24879055

[15] Leclere M, Lavoie-Lamoureux A, Joubert P, Relave F, Setlakwe EL, Beauchamp G, et al Corticosteroids and antigen avoidance decrease airway smooth muscle mass in an equine asthma model. Am J Respir Cell Mol Biol. 2012; 47: 589–596. 10.1165/rcmb.2011-0363OC 22721832

[16] Abraham G, Zizzadoro C, Kacza J, Ellenberger C, Abs V, Franke J, et al Growth and differentiation of primary and passaged equine bronchial epithelial cells under conventional and air-liquid-interface culture conditions. BMC Vet Res. 2011; 7: 26 10.1186/1746-6148-7-26 21649893 PMC3117700

[17] Franke J, Abs V, Zizzadoro C, Abraham G. Comparative study of the effects of fetal bovine serum versus horse serum on growth and differentiation of primary equine bronchial fibroblasts. BMC Vet Res. 2014; 10: 119 10.1186/1746-6148-10-119 24886635 PMC4040117

[18] Shibeshi W, Abraham G, Kneuer C, Ellenberger C, Seeger J, Schoon H, et al Isolation and culture of primary equine tracheal epithelial cells. In Vitro Cell Dev Biol Anim. 2008; 44: 179–184. 10.1007/s11626-008-9099-8 18594938

[19] Moll R, Divo M, Langbein L. The human keratins: biology and pathology. Histochem Cell Biol. 2008; 129: 705–733. 10.1007/s00418-008-0435-6 18461349 PMC2386534

[20] Holgate ST, Holloway J, Wilson S, Bucchieri F, Puddicombe S, Davies DE. Epithelial-mesenchymal communication in the pathogenesis of chronic asthma. Proc Am Thorac Soc. 2004; 1: 93–98. 10.1513/pats.2306034 16113419

[21] Goto Y, Noguchi Y, Nomura A, Sakamoto T, Ishii Y, Bitoh S, et al In vitro reconstitution of the tracheal epithelium. Am J Respir Cell Mol Biol. 1999; 20: 312–318. 10.1165/ajrcmb.20.2.3062 9922223

[22] Osei ET, Noordhoek JA, Hackett TL, Spanjer AI, Postma DS, Timens W, Brandsma CA, Heijink IH. Interleukin-1α drives the dysfunctional cross-talk of the airway epithelium and lung fibroblasts in COPD. Eur Respir J. 2016; 48: 359–369. 10.1183/13993003.01911-2015 27418555

[23] Bogdanowicz DR, Lu HH. Studying cell-cell communication in co-culture. Biotechnol J. 2013; 8: 395–396. 10.1002/biot.201300054 23554248 PMC4230534

[24] Papazian D, Würtzen PA, Hansen SW. Polarized Airway Epithelial Models for Immunological Co-Culture Studies. Int Arch Allergy Immunol. 2016; 170: 1–21. 10.1159/000445833 27240620

[25] Crosby LM, Waters CM. Epithelial repair mechanisms in the lung. Am J Physiol Lung Cell Mol Physiol. 2010; 298: L715–L731. 10.1152/ajplung.00361.2009 20363851 PMC2886606

[26] Nolte SV, Xu W, Rennekampff HO, Rodemann HP. Diversity of fibroblasts—a review on implications for skin tissue engineering.Cells Tissues Organs 2008; 187: 165–76. 10.1159/000111805 18042973

